# Solar energy for electricity and fuels

**DOI:** 10.1007/s13280-015-0729-6

**Published:** 2015-12-14

**Authors:** Olle Inganäs, Villy Sundström

**Affiliations:** Biomolecular and Organic Electronics, IFM, Linköpings Universitet, 58183 Linköping, Sweden; Chemical Physics, Lund University, P.O. Box 124, 22100 Lund, Sweden

**Keywords:** Photovoltaics, Artificial photosynthesis, Photocatalysis, Solar fuel, Photosensitizers, Photocatalysts

## Abstract

Solar energy conversion into electricity by photovoltaic modules is now a mature technology. We discuss the need for materials and device developments using conventional silicon and other materials, pointing to the need to use scalable materials and to reduce the energy payback time. Storage of solar energy can be achieved using the energy of light to produce a fuel. We discuss how this can be achieved in a direct process mimicking the photosynthetic processes, using synthetic organic, inorganic, or hybrid materials for light collection and catalysis. We also briefly discuss challenges and needs for large-scale implementation of direct solar fuel technologies.

## Introduction

**Fig. 1:**
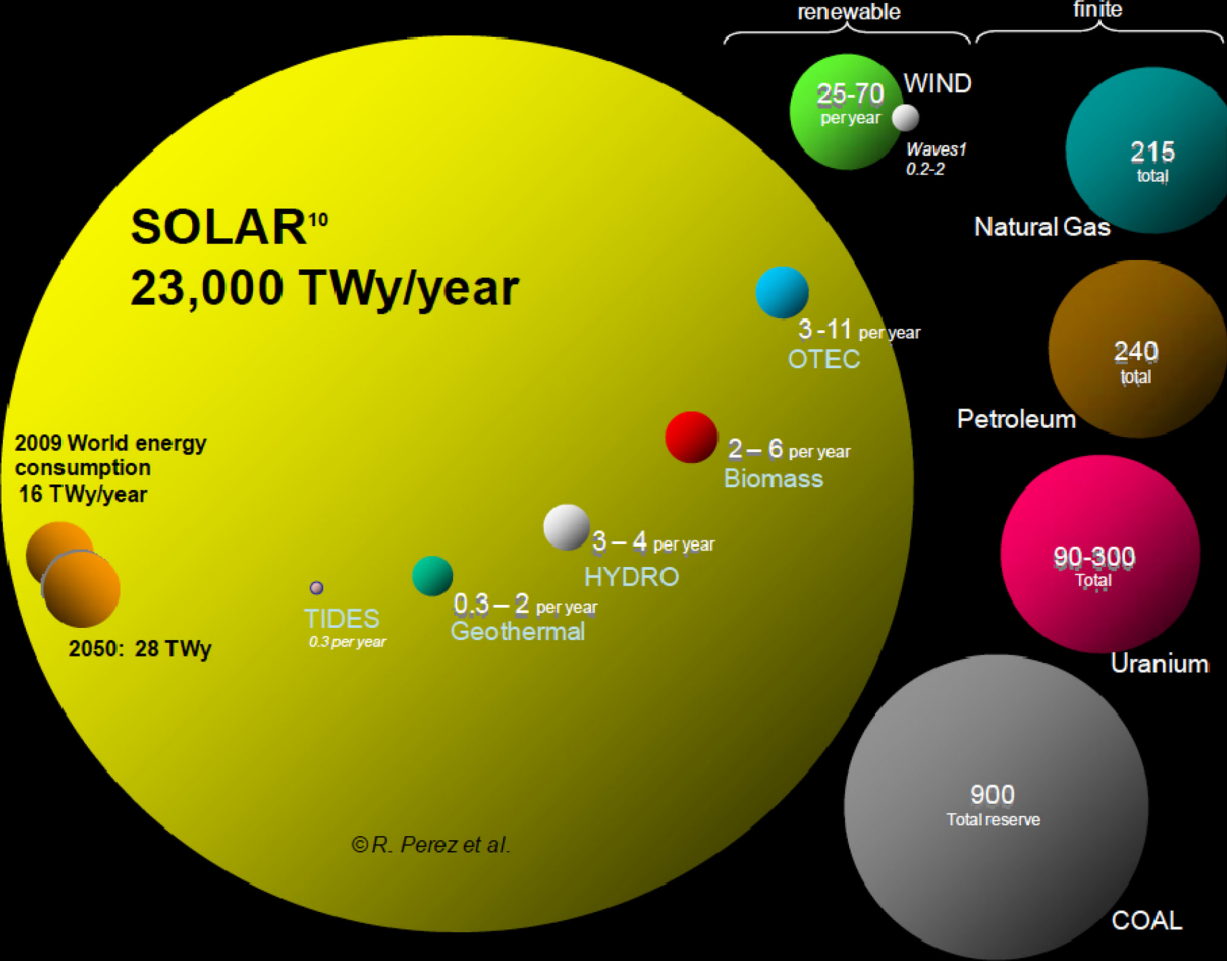
A comparison of finite and renewable planetary energy reserves measured in TW/years. Total recoverable reserves are shown in TWy for the finite resources, and yearly potential for the renewables. OTEC = Ocean thermal energy conversion. One TWy is 8760 TWh. This figure does not include shale gas or shale oil nor the energy available in methane hydrate. Source: Perez and Perez (2009)

Renewable energies are still dominated by bio-energy and hydro-energy. This technology is well developed and more energy can be produced but are inherently limited (Fig. 1). Vegetation exists on some 80 million km^2^ with an average net bio-energy production of around 0.1 W/m^2^. Higher production would require active nitrogen. Hydro-energy is even more limited.

Photovoltaic electricity from solar cells has undergone a rapid development and is rapidly being used both in minor private systems as well as in large-scale installations connected to the national grids. Wind energy has also reach maturity and undergoes presently a rapid worldwide implementation. The main challenge with the renewable energy is the intermittency requiring major storage or large-scale integration.

This article focuses on solar energy, identifying the need for breakthroughs in more efficient ways to produce (i) electricity from more powerful and cost efficient solar cells (ii) the possibility of direct conversion of solar energy into fluid (such as ethanol or methanol) or gas forms (methane of hydrogen), and (iii) the need for producing significantly higher bioconversion including breeding of special plants and genetic engineering of cyanobacteria.

The annual flow of energy from the sun dwarfs all other non-renewable energy flows and stocks, and is several orders of magnitude above what humankind needs (Fig. 1). Very major secondary flows of solar energy are concentrated by the thermal engine of the atmosphere, supplying flow of water for hydropower, and flow of air for wind power. From inside the Earth, geothermal heat is delivered from nuclear processes. All these are very small by comparison to the solar influx. The solar influx over land on a horizontal surface varies considerably between different regions and with the diurnal and annual cycle. The most favorable areas are in the subtropics where values can reach almost 300 W/m^2^ in annual average, while in parts of northern Europe values can be as low as around 50 W/m^2^ with very low values during winter. The diurnal variation of the solar energy influx matches somewhat but not perfectly the activity patterns of societies. There is therefore a great need to store solar derived energy as electrical or chemical energy, to be used at some later time.

## Photovoltaic

### Current scientific status

**Fig. 2:**
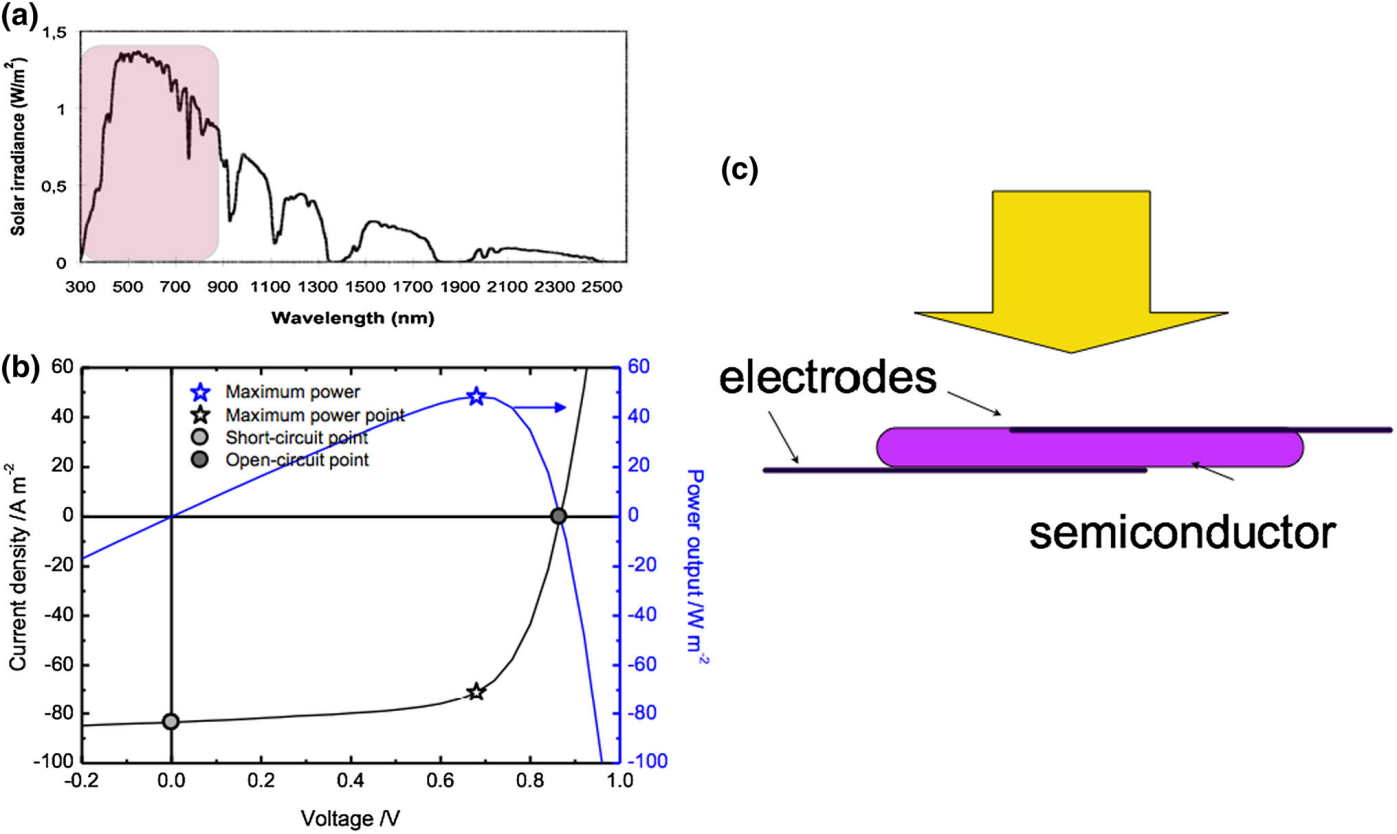
**a** The solar radiation arriving at the surface of the earth is distributed over different wavelengths of light, with tails extending far out in the infrared and invisible region. With semiconductor materials, light with energy higher/wavelengths shorter than the band gap of the material can be absorbed (shadow in graph), but none below the band gap. **b** The semiconductor is contacted with electrodes and exposed to solar light. Photocurrent *J*_sc_ from the devices depends on absorption and charge generation in the active material; the photo-voltage *V*_oc_ basically depends on the materials. **c** The current–voltage curve shows both these parameters, and the device is used to deliver electricity at the maximum power delivery point, indicated with a *star*

Photovoltaic devices generate electrical power upon illumination (Fig. 2). The electrical current is generated as photons from sunlight are absorbed and charges are generated in a semiconductor material. Semiconductors such as silicon absorb a large fraction of sunlight, but below the absorption edge (band gap) of the semiconductor, no absorption occurs. This controls the photocurrent and thus the electrical power of the devices, in combination with the voltage which is influenced by very basic physics and materials science parameters.

The science of photovoltaic is a mature field. Novel photovoltaic materials arrive, and scientists later learn how to accommodate these novel phenomena to the scientific terminology of photovoltaics. The thermodynamic limitations analyzed by Shockley and Queisser (S–Q) in (1961) resulted in a maximum efficiency of slightly over 33 % in a single-junction device using a band gap of 1.1–1.4 eV. These limitations are due to heat losses from high energy photons above the single-band gap, and from loss of photons not absorbed below the band gap. It is also based on the equilibrium flux of photons to the photovoltaic device from the sun, and the flow of photons to the universe from the photovoltaic device. This is a description that is valid for well established materials, like crystalline and poly-crystalline silicon-based photovoltaic, but also for amorphous inorganic semiconductors in thin film format, as well as newer organics in wet or dry state conditions, and new hybrid organic–inorganic materials. Though the science is mature, the implications of this science have not always been fully acknowledged. The recent improvement, to 28.8 % power conversion efficiency (PCE), of high performance gallium arsenide (GaAs) single-junction solar cells, has been accomplished by photonic engineering, to manipulate the balance between the incoming and outgoing photon flux, with the reward coming as voltage from the cell (Miller et al. 2012).

Improved energy conversion is found in multi-junction solar cells, or tandem solar cells, where the heat and transmission losses are minimized by use of two, three, or more different materials of different band gaps, and connecting these devices in electrical series. These are very high performance solar cells, with power conversion efficiencies up to 44.7 % under the direct sun spectrum at very high concentrations. These solar cells are mostly used in space, or under concentrated solar light on earth. The delicate growth of several compound semiconductors on top of each other to create these structures results in a higher material and processing cost. Therefore, these solar cells are mostly relevant together with concentrating optical elements, and thus require high performance, low-cost concentrators, which only work well in direct sunlight. Such concentrator systems are today becoming more widely available and applications in the multi MW range have been realized.

Attempts to use the part of the solar spectrum found in the infrared and far infrared, carrying 50 % of the solar energy, for photovoltaic energy conversion is of relevance, in particular in combination with tandems. For single-band gap devices, the optimum band gap is 1.1–1.4 eV, and the lower energy part of the solar spectrum is sacrificed.

### Current technological developments

#### Developments of silicon photovoltaic markets and technology

The goal of 0.4 €/W_p_ for photovoltaic (PV) modules is close to appear in the silicon photovoltaic market, where the wholesale price is now 0.5 €/W_p_, and where the financial difficulties of most companies indicates that variable costs in their production are c. 0.4 €/W_p_. The dramatic reductions of silicon PV sales prices (to 20 % during the last 5 years) are mainly due to market pressure resulting from oversupply, increased production volumes leading to cost reduction by scale, and to some degree also due to technological advancement. Extrapolating that pace of technological improvement in the near future, short-term market and technology analysis indicate that by year 2017 the production cost of 0.3 €/W_p_ will be reached. The learning curve of silicon PV may, however, decelerate as sales prices go so low that new technology cannot be acquired and financed, or by reaching the technology limits given by the requirement for crystalline layers with – due to the indirect band gap of silicon—large thickness.

However, already today photovoltaic electricity generation cost can be as low as 12 €cents/KWh in countries like France or Germany and 8 €cents/KWh in the south of Europe. Within the next decade solar electricity from photovoltaics is likely to become the cheapest form of renewable energy in most parts of the world. Silicon is an established photovoltaic material, it is widely available, non-toxic and it is likely to dominate the market for the foreseeable future. However, production cost of electricity is not the only element, as the electricity system cost represents typically 40–60 % of the final cost.

This major cost reduction of silicon PV puts significant pressure on all alternative technologies like thin film inorganic photovoltaics (e.g., a-Si, CdTe, CIGS) which lost in market share during recent years, but improved somewhat in record performance numbers. All PV technologies in the future will have to approach higher conversion efficiencies to reduce the contribution of all other module components. Whether there is a place for low efficiency, low-cost solar cells to compete with silicon photovoltaic is now more doubtful than 5 years back, as the cost of silicon has dropped.

The combination of silicon PV with compound semiconductors (thin film III-V compounds or nanowires) in hybrid tandem photovoltaic promises big improvements of power conversion efficiencies, and may be one of the most attractive pathways for rapid improvement in PCE. Also these approaches can benefit from the advances in silicon photovoltaic like lower material costs and enhanced manufacturing technologies.

#### Hybrid organic/inorganic solar cells

High performance dye sensitized solar cells, where an organic compound in contact with a high band gap inorganic semiconductor (e.g., TiO_2_, ZnO, ZrO_2_) generates charge upon photon absorption, still require a redox liquid electrolyte for best performance. Alternative redox design using cobalt compounds has improved efficiencies to 13 %. Solid state versions of the dye sensitized solar cells have lower PCE. A new inorganic absorber, a perovskite based on lead halogen compound first used for dye sensitized solar cells, has rapidly demonstrated that this compound can be used at its best (18 % power conversion efficiency) in thin film geometries (Jeon Jeon et al. 2015), as optical absorption and electronic transport is sufficient in this material; the advantages of the dye sensitized cells are thereby removed for this absorber.

#### Solid state organic solar cells

Development of organic donor/acceptor-based photovoltaic (OPV), where donor and acceptor are typically organic molecules or polymers, is accelerating, with best efficiencies now at 12 % (Heliatek 2012; Mitsubishi 2013). The photo-voltage from these materials is controlled by a charge transfer state generated at the interface between the donor/acceptor (Vandewal et al. 2009). Though the basic physics of these photovoltaic materials is now somewhat mature, there is still a large room for improvement by chemical design. With improvement of 1 % unit per year, and limits to PCE still above 20 %, there is reason to expect 15–20 % PCE as an outcome of novel materials.

As the active materials in OPVs are carbon based, these are generally very scalable materials. The production methods for OPV include printing from solvents or evaporation of molecules through vacuum. Both are amenable for large area deposition, with somewhat different potential speed of production and capital costs of equipment. Printing is the faster and cheaper, and rapidly scalable to produce large areas of photovoltaic modules on the global scale.

For both OPV and dye sensitized cells, they operate well under low lights, high temperatures and considerably improve the light harvesting compared to silicon devices. The extra yield of almost a 1/3 from an organic-based module will give the same amount of kWh from a 15 % organic module as compared to a 20 % silicon module.

In the comparison of early organic photovoltaic systems, which have no market share yet, issues of lifetime and module efficiency are largely unresolved. Whether they will be sufficient to compete with mature silicon photovoltaic is not clear, and they will most probably not enter into power plants but rather be used in building integration.

### Needs for breakthroughs

#### Scalability of semiconductor materials

Silicon- or carbon-based materials, which can be scaled up, deliver 25 % (Si) and 12–13 % (carbon based) power conversion efficiency in best lab devices. Several of the competing thin film technologies suffer from a dependence on rare or toxic materials which reduces their potential to play a significant role when photovoltaics will be used in large-scale systems. Therefore, the use of earth abundant materials or efficient recycling concepts will be important for large-scale deployment of the technologies. Removing materials limitations due to less abundant elements (for active materials e.g., indium, gallium, tellurium, for current collectors silver) by substitution with more abundant elements (e.g., zinc, sulfur or copper, aluminum) will be a general and critical target.

#### Decreasing the energy payback time of solar cells

Measures and means needed

- Reducing the energy input in photovoltaic modules production. Low-temperature processes, substrates with low embodied energy, repeated use of substrates for growth of high quality materials/devices, closure of the materials cycle during the life cycle of devices;
- Extending the technical lifetime of high performance photovoltaic by improved encapsulation and protection.

### Materials design

- Designing the electronic properties of carbon-based materials by understanding and controlling the path from single molecular structures via nano-morphology to film growth. A prime example is bulk heterojunctions in organic solid state solar cells which need a nanometer control of morphology in the key active layer.

Areas where scientific and materials breakthroughs may be possible and of potential importance

- Multiple-band gap tandem solar cells incorporating scalable materials;
- Light concentration through far field optics for multiple-band gap materials;
- Nanophotonics in dielectrics and semiconductors for light-matter coupling, light trapping/ light management;
- Plasmonics for light-matter coupling, to direct energy to the semiconductor structure in photovoltaic devices;
- Combination with solar fuel generation, e.g., hydrogen generation by photovoltaic layers with catalyst electrodes.

### Identification of research needs of the coming decades

#### Nanophotonic strategies for light trapping in thin structures

The well established Yablonovitch limit for light trapping in the limit of geometrical optics is not relevant when arriving at thin film structures that must be analyzed with the wave optics approach. This goes for thin film devices with extremely thin absorbers, and new strategies for optical compression of solar light into such thin absorbers have been proposed. Considerable experimental developments are necessary to exploit such strategies, shaping, and integrating thin film absorbers in a benign optical environment.

#### Transparent electrode materials, not relying on elements of low abundance

- Carbon based or abundant metal oxide transparent conductors for electrodes;
- New combinations of device/module assembly to generate lower currents and higher voltages for decrease of losses in photovoltaic modules.

### Beyond S–Q limits?

The perennial quest for ways of moving beyond the Shockley–Queisser limits for single- and multiple-band gap absorbers in photovoltaic devices may require new approaches for generating electricity. When developing hybrid approaches based on high performance semiconductors, the added energy conversion with novel materials and structures may include up-conversion. Without a band gap alternative physical mechanisms must be found. Current developments in thermoelectrics points to new combinations of photovoltaics and thermoelectrics; optical antennas and rectennas show interesting avenues for creating absorbing structures somewhere between geometric antennas and molecular/elemental semiconductors with defined band gaps.

## Solar fuels: Artificial photosynthesis

Direct conversion of solar energy into a fuel mimicking the catalytic processes of photosynthesis would be a way to solve the problem of storing solar energy. A direct process would minimize the number of steps needed from light to fuel, and therefore potentially allow for high conversion efficiencies. The raw material for the (storage) fuel is an important key aspect for the solar fuels. The desirable raw material must be essentially inexhaustible, cheap, and widely available. Most scientists target water as the raw material. Processes in which water is split (oxidized) into its constituents by solar energy can become large contributors to shift away from fossil fuels on a global scale.

The target fuel is another important issue. Many scientists target hydrogen as the solar fuel. When water is used as starting material this is natural for scientific reasons. However, the transition to a hydrogen-based economy is not easy. The hydrogen technologies have their own hurdles to overcome and technological issues to tackle before hydrogen can become a worldwide spread fuel. An alternative is to use CO_2_ itself as a second raw material (together with water) to create a carbon-based solar fuel. This might have lower technological problems on a shorter term but will not remove CO_2_ from the energy cycle (see Fig. 1). In addition, some of the science involved in developing methods for fuel production is most likely more difficult than for hydrogen production.

**Fig. 3:**
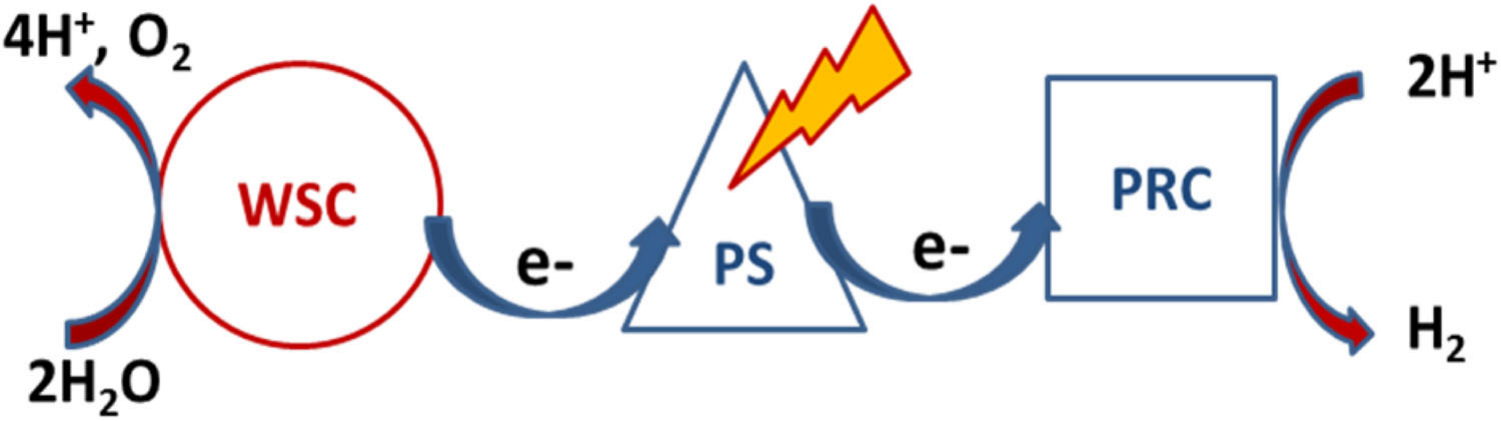
Schematic picture of an artificial photosynthesis assembly consisting of a light collecting photosensitizer (PS), a proton reducing catalyst (PRC), and a water splitting catalyst (WSC)

The envisaged key components of an assembly for production of a solar fuel (H_2_) using water as raw material are illustrated in Fig. 3 (Hammarström and Hammes-Schiffer 2009; Cogdell et al. 2013). A photo-sensitizer (PS) absorbs the light from the sun and delivers energized electrons to the proton reducing catalysts (PRC) that generates the fuel, H_2_. The electrons delivered by the sensitizer are replenished by oxidizing (splitting) water with the help of a water splitting catalyst (WSC). In an operating device the WSC and PRC functions would most likely be driven by different sensitizers and physically separated and located on different photo-electrodes in contact with water.

### Predicted power conversion efficiencies: Direct processes for solar fuel

Today, no complete device for (direct) solar fuel generation exists, but sensitizers and catalysts that perform the individual reactions have been developed. Nevertheless, a maximum theoretical Shockley–Queisser (S–Q) efficiency of ~30 % for (direct) solar fuel generation (hydrogen and oxygen from water) can be estimated for a single-band gap sensitized process and assuming a realistic value (~0.15 V) for the over-potential (Hanna and Nozik 2006; Blankenship et al. 2011). It is foreseeable that the time curve for the development of solar fuel generation devices, with respect both to efficiency and cost, will be faster than for the different PV technologies, because the development to some extent will benefit from progress already made in neighboring fields. The most important being the scientific level in light management, electron and proton transfer, material and nano-science, biochemical, and natural photosynthesis. Technological advances in characterization methods, such as time resolved spectroscopy of many kinds, synchrotrons and new laser techniques, are also vital. Sensitizers and catalysts available today frequently contain elements that are scarce, expensive, or toxic. For large-scale commercial implementation of solar fuels produced by artificial photosynthesis technologies materials based on abundant and environmentally benign elements have to be developed, which most likely will take an additional 10 years or more. This restriction is analogous to PV technology limitations.

In comparison, for solar fuel generation relying on a combination of PV and electrolysis, the thermodynamic limits are given by the S–Q treatment in combination with conditions for electrolysis. For the case of photoelectrolysis, with production of H_2_ and O_2_, the over potentials for oxidation of water to oxygen and reduction of protons to hydrogen must be added to the thermodynamic potential of 1.23 V for dissociation. Driven by solar cells, this requires more than one cell, added in series, to generate sufficient voltage for electrolysis. With electrolysis having a quantum efficiency of 80–90 %, and a single-band gap cell of 33 % power conversion efficiency, the maximum efficiency from photon to hydrogen may be limited to ≈30 %. The “artificial leaf” construction of Nocera (2012), comprising a triple junction amorphous silicon photovoltaic cell interfaced with hydrogen- and oxygen-evolving catalysts made from a ternary alloy (NiMoZn) and a cobalt_phosphate cluster (Co-OEC), respectively, is an example of where this technology stands today. The reported overall efficiency of this device is 2.5 %.

### Methods to produce direct solar fuels

At present there is no direct process that works on any technological scale. The large potential in the direct processes make them nevertheless attractive future technologies and their development is expected to be require heavy research initiatives in several fields.

As mentioned above and illustrated in Fig. 3, the key steps for direct solar fuel production mimicking photosynthesis are two catalytic reactions, oxidation of water and reduction of a substrate to produce the fuel. If the substrate is protons a relatively well established catalytic process yields the hydrogen fuel. A carbon-based fuel, for example, an alcohol, might be easier to use, but is probably more difficult to make since it would involve less established catalytic processes. A highly critical aspect is that the catalysts should be made from earth abundant metals like cobalt, manganese, iron and nickel, while scarce elements like iridium, indium, palladium, platinum, rhenium, and ruthenium are not available in amounts large enough to allow development of processes and materials of a scale sufficient to replace fossil fuels.

Molecular and semiconductor nanostructure processes. The light-driven catalyst can be molecular (Hammarström and Hammes-Schiffer 2009; Sundström 2009; Nocera and Guldi 2009; Kamat and Bisquert 2013) or non-molecular (Cook et al. 2010; Poddutoori et al. 2011; Vagnini et al. 2013; Joya et al. 2013). The physical limitations are equal and the scientific problems are of equal magnitude. The development of catalysts that can assist the light-driven oxidation of water is the main research problem. The capture of solar energy and the formation of hydrogen are easier to achieve. Systems combining the two reactions (light-driven water oxidation and hydrogen formation) have the highest potential with respect to solar energy to fuel conversion of all systems envisioned today (Fig. 3).

The catalysts and maybe the entire system for artificial photosynthesis can be entirely molecular in nature. They can thereby be varied through small, deliberate synthetic modifications to improve and fine-tune their properties. They are also amenable to studies with high-level molecular or kinetic spectroscopy. Thereby, the catalytic process can be followed and understood to a very detailed level. A special angle to this research involves biomimetic approaches. Here, knowledge and chemical principles from extremely efficient enzymes are applied in entirely synthetic systems for artificial photosynthesis. A successful scientific example involves the development of di-iron catalysts for the reduction of protons to hydrogen. These are designed from deep knowledge about the structure and function of so called hydrogenase enzymes and there are recent examples of very efficient catalysts in the category (Tamagnini et al. 2007). Another example involves mimics of the natural photosynthetic reaction centres, with both catalysis and light harvesting in future artificial photosynthesis devices (Magnuson et al. 2009).

In non-molecular systems the light-driven catalysis occurs on metal surfaces, semiconductors or nanostructured carbon-based materials while the catalysts involved for water splitting are often cores of metal oxides, sometimes doped with other metals. A severe limitation is that systems many times are based on catalysts made from scarce and expensive metals. Another disadvantage when compared to the molecular systems is that it is much more difficult to study the mechanism for the reactions involved. An important advantage is that many non-molecular systems are seemingly sturdier against degradation and inactivation while most molecular systems studied to date are unstable and easily break during illumination. It is not obvious that this situation will always prevail when more functional systems have been better characterized. An analogy is the Grätzel cell where the individual parts are fairly unstable but the integrated system is very stable.

A rapid development involves ideas where molecular and non-molecular systems are mixed. They are sometimes known as hybrid systems and the science is very broad and collaborative, involving several scientific fields. Here, the solar energy capture system is semiconductor based or made from some other nanotechnology while the chemistry is carried out by linked molecular catalysts. These catalysts are then made from abundant materials like cobalt, iron, manganese, or nickel similar to the “purely” molecular systems described above. Often catalysis is driven by light via incorporation of a photo-sensitizer between the semiconductor and the catalyst. It is not unlikely that these mixed hybrid systems will become dominant in research and maybe technology since they combine advantages from both fields.

Thermochemical cycles. A totally different technology is the employment of thermal processes (Pagliaro et al. 2010) for solar fuels production, which involve generation of very high temperatures in closed environments to split water into its constituents directly. This results in a solar fuel when the high temperature is achieved in a reaction vessel in a solar tower by concentration of solar energy in a heliostat. The technology represents interesting engineering and physical science and is very demanding technically involving very high temperatures and huge systems like heliostats. They are mainly suitable to very sunny locations.

### Direct solar fuel needs for breakthroughs

#### Efficient photo-sensitizers and photocatalysts

For large-scale implementation of direct solar fuel technologies, water will necessarily be the ultimate electron source for reducing the fuel-producing substrate, protons, CO_2_, etc. New and highly efficient catalysts for water oxidation and fuel generation are urgently needed, as well as sensitizers based on abundant elements:

- Photo-sensitizers frequently contain noble metals (ruthenium), which have to be replaced with cheap and abundant elements. Iron is one example but dyes based on Fe and similar transition metals have unfavorable properties for solar energy conversion, necessitating scientific breakthroughs. At the same time fully organic dyes are appearing. Thus, it can be expected that sensitizers suitable for most applications and for large-scale implementation are likely to appear in not too distant future.
- To achieve highly efficient catalysts for water oxidation and hydrogen (liquid fuel) generation is a considerably more difficult and complex problem. Catalysts may be either molecular complex or solid state (e.g., metal oxide) based. Understanding the mechanisms of O-O and H-H bond formation is key to the development of efficient catalysts. Most of today’s catalysts have low efficiencies and are frequently based on scarce or noble metals, making them unsuitable for large-scale commercial implementation. Here, scientific breakthroughs are needed, both concerning efficiency and developing new catalysts based on abundant and cheap elements. Proof-of-concept catalysts with high efficiencies, based on metals like ruthenium, are likely to appear within the next 10 years, but catalysts suitable for commercial applications most likely will take considerably more time.

#### Liquid fuels

Liquid solar fuels would facilitate the use of existing infrastructure for the utilization of the fuel. By combining the protons and electrons released as a result of water oxidation with CO_2_ in a catalytic process, alcohols or other liquid fuels could be synthesized. This is a scientifically challenging task and there are today no good catalysts for CO_2_ reduction. Another problem is that the concentration of CO_2_ in the atmosphere is low, necessitating an energy-consuming concentration step, or coupling of the solar fuel production to CO_2_-emissions from e.g., a fossil-fuel power plant or a CCS plant (see MacElroy 2016).

#### Functional devices for direct solar fuel production

No complete device for (direct) solar fuel production exists. New concepts to assemble sensitizers and catalysts into a functional device producing a fuel through light-driven water splitting have to be developed. One possibility that is considered within the field is to attach sensitizer-catalyst assemblies to a nanostructured electrode surface and placing these electrodes in separate compartments, one for fuel (hydrogen) generation and the other for oxygen evolution. Here, suitable electrode systems have to be developed—methods for sensitizer-catalyst assembly attachment to the electrode must be developed, and electrochemical properties of catalysts and electrode materials matched. Considering that this development is still in its scientific infancy, it will probably take more than 10 years until efficient proof-of-concept systems have been developed and considerably more time until commercially suitable devices are available.

#### Scalability of photo-sensitizers and catalysts

Like for PV, large-scale implementation of solar fuel technology requires cheap and earth abundant materials. Today’s molecular or semiconductor photo-sensitizers and photocatalysts often contain scarce, noble (and expensive), or toxic metals, which obviously need to be replaced by more abundant and benign elements. This may take a considerable amount of time and effort, since the photophysical, photochemical, and catalytic properties of materials based on the abundant elements are frequently very different from those of the metals used today. It will require extensive development work to achieve requested properties and efficiencies of the abundant materials.
